# Explainable artificial intelligence and microbiome data for food geographical origin: the Mozzarella di Bufala Campana PDO Case of Study

**DOI:** 10.3389/fmicb.2024.1393243

**Published:** 2024-06-03

**Authors:** Michele Magarelli, Pierfrancesco Novielli, Francesca De Filippis, Raffaele Magliulo, Pierpaolo Di Bitonto, Domenico Diacono, Roberto Bellotti, Sabina Tangaro

**Affiliations:** ^1^Dipartimento di Scienze del Suolo, della Pianta e degli Alimenti, Università degli Studi di Bari Aldo Moro, Bari, Italy; ^2^Istituto Nazionale di Fisica Nucleare, Sezione di Bari, Bari, Italy; ^3^Dipartimento di Agraria, Università degli Studi di Napoli Federico II, Naples, Italy; ^4^Dipartimento Interateneo di Fisica M. Merlin, Università degli Studi di Bari Aldo Moro, Bari, Italy

**Keywords:** explainable artificial intelligence, machine learning, microbiome, food origin, PDO

## Abstract

Identifying the origin of a food product holds paramount importance in ensuring food safety, quality, and authenticity. Knowing where a food item comes from provides crucial information about its production methods, handling practices, and potential exposure to contaminants. Machine learning techniques play a pivotal role in this process by enabling the analysis of complex data sets to uncover patterns and associations that can reveal the geographical source of a food item. This study aims to investigate the potential use of explainable artificial intelligence for identifying the food origin. The case of study of Mozzarella di Bufala Campana PDO has been considered by examining the composition of the microbiota in each samples. Three different supervised machine learning algorithms have been compared and the best classifier model is represented by Random Forest with an Area Under the Curve (AUC) value of 0.93 and the top accuracy of 0.87. Machine learning models effectively classify origin, offering innovative ways to authenticate regional products and support local economies. Further research can explore microbiota analysis and extend applicability to diverse food products and contexts for enhanced accuracy and broader impact.

## 1 Introduction

With the burgeoning demand for high-quality, region-specific products, the need to ensure the origin and treceability of food products plays a pivotal role in ensuring authenticity, quality, and safety in the global food supply chain (Gallo et al., 2021). The concepts of food traceability and origin are closely interlinked and hold pivotal significance in ensuring food safety and transparency throughout the production process but also supports local economies and encourages sustainable agricultural practices. They are integral in guaranteeing that foods are safe, genuine, and adhere to quality standards. Traceability refers to the ability to follow the journey of a product along the entire supply chain, encompassing detailed information about its production, processing, packaging, distribution, and sale (del Rio-Lavín et al., 2023). On the other hand, the origin of food products indicates the specific location where they were cultivated, manufactured, or processed. Understanding the origin of a food item is essential for various reasons, including ensuring its safety, quality, and sustainability. Presently, determining the origin of a food product relies on diverse methods and tools. Collaboration among producers, distributors, and other stakeholders in the supply chain is crucial to ensuring transparency and accuracy in disclosing the origin of food products (Corallo et al., 2020). Some food products may acquire origin certifications, such as the Protected Designation of Origin (PDO) in Europe or other regional certifications, which verify that the product originates from a specific geographical area and complies with designated standards (Badia-Melis et al., 2015). Analyzing the intricate ecosystem of microorganisms inhabiting food, known as the food microbiota, can be a useful tool for understanding the safety, quality, and characteristics of food products of foods. This diverse microbial community, comprising bacteria, fungi, and viruses, is influenced by various factors such as geographical location, production methods, and processing techniques. A fundamental aspect of harnessing the food microbiota for product origin lies in its dynamic composition, which reflects the unique environmental conditions and production practices of each food item. By scrutinizing the microbiota composition of food samples, distinctive microbial signatures indicative of their origin or production environment can be discerned. Recent advancements in molecular biology and sequencing technologies have revolutionized our ability to characterize the food microbiota with unprecedented precision and speed. High-throughput sequencing methods, including next-generation sequencing, facilitate rapid and accurate identification of microbial species present in food samples (Reuter et al., 2015). Comparative analysis of microbiota profiles among different food samples enables the identification of subtle variations that serve as valuable markers for product origin. Specific microbial strains or community structures may be linked to particular regions or production facilities, offering distinctive identifiers for food products. Moreover, the food microbiota serves as a sentinel for monitoring food quality and safety along the supply chain (Guidone et al., 2016). Alterations in microbial composition or abundance can signal potential contamination or spoilage incidents, enabling prompt interventions to mitigate risks and uphold food safety standards. In addition to conventional laboratory techniques, emerging methodologies such as metagenomics and metatranscriptomics provide comprehensive insights into the food microbiota. These cutting-edge approaches enable holistic analysis of all microbial genetic material within a sample, facilitating deeper understanding of microbial dynamics and functions (Cao et al., 2021). The use of machine learning in food classification and origin represents a significant step forward in ensuring the safety and authenticity of food products. Firstly, machine learning enables the development of predictive models that can differentiate between different types of foods based on specific characteristics. By leveraging machine learning algorithms, it becomes possible to process vast amounts of data, including information on production practices, environmental factors, and biochemical compositions, to accurately predict the origin of a food product. For example, using data from chemical, sensory, or genetic analyses, models can be trained to recognize the presence of contaminants or identify the geographical origin of a food. Furthermore, the application of machine learning to food classification offers numerous opportunities to enhance food safety, ensure product authenticity, and optimize the identification of food origin. The integration of machine learning and microbiota offers an innovative approach to understanding the complexity of interactions between the microbiome and food. By analyzing microbiome data using machine learning algorithms, it is possible to identify patterns and associations that can be valuable for enabling the develop preventive strategies to reduce risks and improve the nutritional quality of foods. The application of machine learning techniques in the field of food microbiota presents multiple opportunities to analyze large amounts of microbiological data, identify patterns and associations between microbial composition and food characteristics, predict food quality and safety, to understanding microbial dynamics and search for solutions to promote health (Bellantuono et al., 2023; Papoutsoglou et al., 2023). Through data analysis and the development of predictive models, crucial challenges in the food industry can be addressed, promoting greater transparency and trust among consumers. Explainable Artificial Intelligence (XAI) algorithms are useful to make artificial intelligence (AI) models understandable and interpretable to humans, because many machine learning and AI models often operate as “black boxes,” making it difficult to understand how and why they produce certain predictions or decisions. The goal of XAI is to provide explanations and insights into the operation of AI models, enabling users to understand the reasons behind their predictions or decisions. This is particularly important in contexts where transparency, accountability, and trust in AI are crucial. In Explainable Artificial Intelligence (XAI), trustworthiness plays a role in ensuring the reliability and transparency of AI models. It refers to the degree of confidence and faith users have in the explanations provided by the model regarding its predictions and decision-making processes. XAI techniques may include SHapley Additive exPlanations (SHAP) analysis that seek to translate the internal workings of AI models into understandable human explanations (Novielli et al., 2024). This research delves into the crucial realm of preserving and authenticating the geographical origin of Mozzarella di Bufala Campana PDO, specifically focusing on the provinces of Salerno and Caserta. The characteristic that will be used for data analysis is the abundance of bacteria present in the microbiota of the samples. This information will be crucial for identifying any patterns or correlations between bacterial composition and the geographical origin of Mozzarella di Bufala PDO. By utilizing data analysis techniques such as machine learning (Monaco et al., 2021; Papoutsoglou et al., 2023), it will be possible to create predictive models capable of accurately classifying the geographical origin of each sample based on microbiota information. This approach will provide a trustworthy assessment of the mozzarella's origins, thereby contributing to food quality and safety.

## 2 Materials

The data utilized in this study, decripted in Table 1 stems from the microbiological analysis of the microbiome of 65 samples of Mozzarella di Bufala PDO originating from 30 dairies in the province of Salerno and 35 dairies in the province of Caserta. These samples underwent thorough examination in the laboratories of the Microbiology Division within the Department of Agricultural Sciences at the University of Naples Federico II. All dairies were located within the PDO area produced traditional Mozzarella di Bufala according to the PDO regulation. Total DNA was extracted using the Qiagen Power Soil Pro kit. Metagenomic libraries were prepared using the Nextera XT Index Kit (Illumina, San Diego, California, United States), then whole metagenome sequencing was performed on an Illumina NovaSeq platform, leading to 2 × 150 bp, paired-end reads. Reads were quality-checked and filtered through Prinseq-lite v. 0.20.4, using parameters “-trim_qual_right 5” and “-min_len 60.” An average of 25 M of paired-end reads were obtained (2 × 150 bp) for each sample. Raw reads were pre-processed and filtered as previously described (De Filippis et al., 2021). Briefly, contamination from host reads was removed using the Human Sequence Removal pipeline developed within the Human Microbiome Project by using the Best Match Tagger (BMtagger) mapping reads against the *Bubalus bubalis* (Mediterranean breed) genome (accession number: GCA003121395.1). Then, non-host reads were quality-filtered using PRINSEQ v. 0.20.4 (Schmieder and Edwards, 2011). Bases having a Phred score < 15 were trimmed and those < 75 bp were discarded. High-quality reads were further processed to obtain microbiome taxonomic profiles using MetaPhlAn v. 4.0 (Blanco-Míguez et al., 2023).

**Table 1:** Description of samples and input variables.

**Type of samples**	**Diary from Campania region**
*n* samples from Salerno	30
*n* samples from Caserta	35
Type of input variables	Microbiome relative abundance
*n* input variables for each sample	139

Our analysis encompasses a diverse set of samples, reflecting the regional diversity of Mozzarella di Bufala PDO production across different dairies in the provinces of Salerno and Caserta. The 65 samples provide a robust dataset for investigating variations in microbial composition, offering valuable insights into the distinctive qualities of Mozzarella di Bufala PDO from different geographic origins. The species abundance data unveils the relative prevalence of microbial species, offering insights into the intricate microbiome of Mozzarella di Bufala PDO. This information is organized in a tabular format, where each row corresponds to a specific sample, and each column represents a distinct microbial species. To enhance our understanding of the origin of each Mozzarella di Bufala PDO sample, we include details about the respective cheese dairy, specifying both the dairy name and its geographic origin. Each sample presents 139 output variables, each representing the abundance of a specific bacterium. In the context of your analysis on the microbiome of Mozzarella di Bufala PDO, these output variables likely reflect the proportions or relative quantities of different types of bacteria present in each sample. The type of bacteria and their relative abundance in each sample could have significant implications for the quality and sensory characteristics of the product. Since many samples have abundance values equal to zero, indicating the absence of the bacteria, a preprocessing step was performed. In this pre-processing step, columns with more than 70% zero values were removed, reducing the total number of columns to 23. In order to conduct a robust analysis, the initial dataset has been strategically partitioned into a validation dataset and a test dataset to. This partitioning is designed to ensure a representative and unbiased evaluation of the models developed during the study (Ibrahimi et al., 2023). The validation dataset consists of 22 samples from the province of Salerno and 33 samples from the province of Caserta. This division allows for the exploration of regional variations within the microbiome of Mozzarella di Bufala PDO, considering the distinctive characteristics of these geographical locations. The validation set was then used to assess three different classifiers through a five-fold cross-validation repeated 20 times (Schaffer, 1993), and the performance of the best classifier (Random Forest, RF) was analyzed. Following that, the trained model was tested on the test dataset, and its performance was evaluated on this separate set of samples.

The independent test dataset, on the other hand, comprises eight samples from Salerno and two samples from Caserta. Notably, these 10 test samples are collected on the same day from the same dairy as the samples present in the validation set. By adopting this partitioning strategy, we aim to develop a model that not only captures the nuances of the training dataset but also demonstrates robust predictive abilities when faced with previously unseen samples.

## 3 Methods

**Figure 1:**
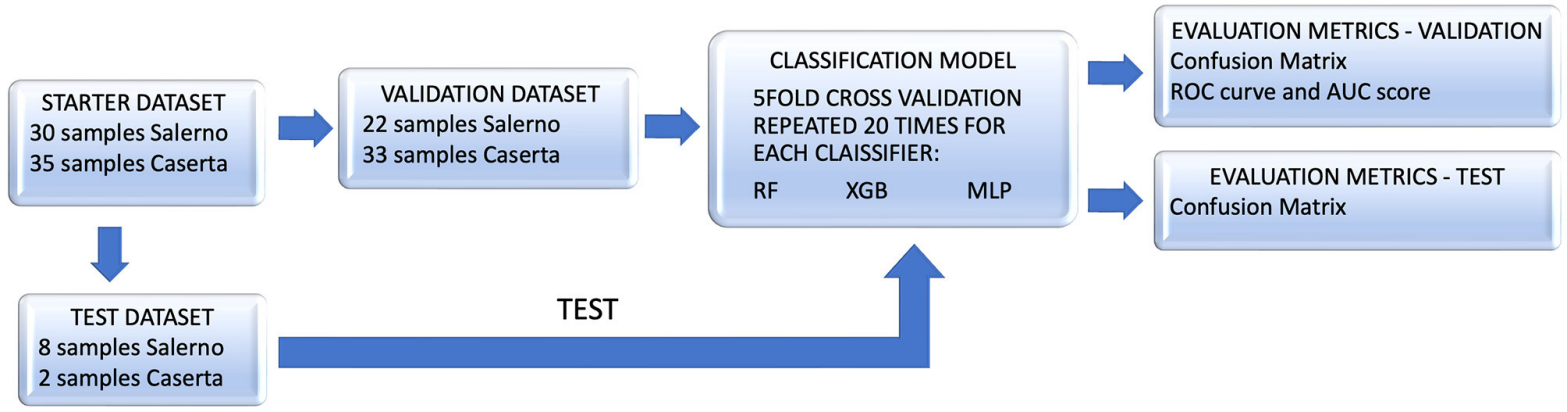
The flowchart outlines the steps of the conducted analysis. The validation set was used to assess three different classifiers through a five-fold repeated 20 times cross-validation, and the performance of the best classifier (Random Forest, RF) was analyzed. Following that, the trained model was tested on the test dataset, and its performance was evaluated on this separate set of samples.

The main steps of our analysis are outlined in the flowcharts in Figure 1. It provides a comprehensive overview of the model's performance during both the training and validation phases, as well as in the subsequent testing phase, allowing for an overall evaluation of its predictive capabilities.

### 3.1 Machine learning based classification

To assess the classification of these samples, three distinct supervised machine learning methods were employed: Random Forest, XGBoost, and Multi-Layer Perceptron (MLP). The identification of the optimal classifier was based on both accuracy and Area Under the Curve (AUC).

#### 3.1.1 Random forest classifier

The Random Forest Classifier represents a sophisticated ensemble learning algorithm within the realm of machine learning (Chaudhary et al., 2016). Envisioned as a confluence of decision trees, it operates on the principle of aggregating predictions from diverse models to augment stability and overall performance. The ensemble is constituted by an assembly of decision trees, each meticulously trained on a distinct subset of the training dataset through the lens of bootstrap sampling a method characterized by its sampling with replacement. The algorithm's efficacy is derived from the varied nature of decision trees. This diversity, arising from the differential subsets of data upon which each tree is trained, mitigates the risk of overfitting, fostering a robust model. In the predictive phase, each decision tree contributes its prediction, and the final class is determined through a majoritarian consensus. This collective decision-making process amplifies the model's resilience and generalization capabilities (Breiman, 2001).

#### 3.1.2 EXtreme gradient boosting classifier

EXtreme Gradient Boosting (XGBoost) is a widely-used machine learning algorithm for regression and classification problems renowned for its prowess in diverse applications, particularly excelling in the realm of structured or tabular data and supervised learning scenarios (Shwartz-Ziv and Armon, 2022). XGBoost has been extensively used in data science and machine learning competitions due to its ability to achieve excellent performance on a wide range of problems and datasets. It's also known for its flexibility and ability to handle large amounts of data. Positioned within the domain of ensemble learning, XGBoost elevates traditional gradient boosting algorithms to new heights. XGBoost typically builds an ensemble of decision trees, where each tree contributes to the final prediction. The combination of multiple trees enhances the model's predictive capabilities. XGBoost supports built-in cross-validation, enabling robust model evaluation and parameter tuning for optimal performance. XGBoost has an high predictive accuracy. By constructing an ensemble of models, each correcting the errors of the others, it can provide more accurate predictions compared to many other algorithms. It also incorporates regularization techniques that help manage the issue of overfitting, keeping the model general and adaptable to new data (Chen and Guestrin, 2016).

#### 3.1.3 Multi-layer perceptron classifier

The Multi-Layer Perceptron (MLP) stands as a sophisticated architecture within the domain of artificial neural networks, prominently featured in the landscape of machine learning. It is distinguished by its layered composition, comprising an input layer, one or more hidden layers, and an output layer. Each layer encompasses interconnected nodes, or artificial neurons, where the transmission of information follows a feedforward trajectory, progressing from the input layer through the hidden layers and culminating in the output layer. In a Multi-Layer Perceptron (MLP), input nodes constitute the initial layer of the neural network and serve as the units through which data is introduced into the system. Each input node represents a specific feature or variable from the dataset intended for model training. The hidden layers are intermediary layers between the input and output layers, responsible for capturing and learning complex patterns and representations within the input data. These layers contribute to the model's ability to discern intricate relationships that may not be immediately apparent in the raw features. Output nodes constitute the final layer of the neural network and are responsible for producing the model's predictions or outcomes. The configuration and characteristics of the output layer depend on the nature of the task, whether it involves classification, regression, or other specific objectives (Ruck et al., 1990).

### 3.2 Evaluation metrics

Evaluation metrics are crucial tools for assessing the performance and effectiveness of machine learning models (Ferrer, 2022). These metrics provide quantitative measures that help quantify how well a model is performing on a given task. The choice of evaluation metrics depends on the nature of the problem (classification, regression, etc.) and the specific goals of the analysis. Here are some commonly used evaluation metrics:

- Accuracy:

The proportion of correctly classified instances among the total instances

(1) $$\begin{array}{l} ACC=\frac{TP+TN}{TP+FP+TN+FN} \end{array}$$

- Sensitivity:

The fraction of true positive predictions out of all actual positive instances

(2) $$\begin{array}{l} SENS=\frac{TP}{TP+FN} \end{array}$$

- Specificity:

Specificity is the proportion of actual negatives correctly identified by the model out of the total number of actual negatives.

(3) $$\begin{array}{l} SPEC=\frac{TN}{FP+TN} \end{array}$$

- Precision:

The fraction of true positive predictions out of all positive predictions

(4) $$\begin{array}{l} PREC=\frac{TP}{TP+FP} \end{array}$$

- Area Under the ROC Curve (AUC-ROC):

The Receiver Operating Characteristic (ROC) curve and Area Under the Curve (AUC) are assessment tools employed to gauge the effectiveness of a binary classification model. The ROC curve presents a graphical depiction of how sensitivity (true positives) and specificity (true negatives) change across various classification thresholds. Essentially, it illustrates the balance between accurately identifying positive and negative instances by the model. The AUC quantifies the overall performance of the model by measuring the area under the ROC curve: a value closer to 1 signifies superior model performance, while a value around 0.5 suggests random classification. In summary, these metrics are vital for evaluating and contrasting the classification ability of binary models (Ozenne et al., 2015).

### 3.3 Explainable artificial intelligence methods

Explainable Artificial Intelligence (XAI) is a crucial aspect in the development of AI systems, focused on making artificial intelligence (AI) models understandable and interpretable to humans. A specific method employed for XAI is the SHapley Additive exPlanations (SHAP) (Arrieta et al., 2020). SHAP values are used to evaluate the impact of individual features on the model's performance, particularly on a validation set. Mathematically, the SHAP value for a specific feature (*j*) is calculated based on the inclusion or exclusion of that feature from the model as:


(5) $$\begin{array}{l} \Phi_{j}\left(x\right)=\underset{F\subseteq S-\left\{j\right\}}{\sum }\frac{|F|!\left(|S|-|F|-1\right)!}{|S|!}\left[f_{x}\left(F\cup j\right)-f_{x}\left(F\right)\right] \end{array}$$

where Φ_*j*_(*x*) represents the SHAP value of feature *j* for the prediction of the model *f* given the input *x*, *S* is the set of all features, *F* ⊆ *S*−{*j*} represents all possible subsets of features excluding feature *j*, $\frac{|F|!\left(|S|-|F|-1\right)!}{|S|!}$ is a weight parameter that multiplies all of the permutations of S! by the potential permutations of the remaining class that doesn't belong to S, while *f*_*x*_(*F*∪*j*) and *f*_*x*_(*F*) denote respectively the model's prediction when feature *j* is added to the subset *F* and when it is absent (Lundberg and Lee, 2017). We also averaged the ten realizations of SHAP values in order to obtain a single representative SHAP vector.

The SHAP value measures how much including feature j changes the model's prediction compared to the prediction without feature j, averaged over all possible combinations of features. Positive SHAP values indicate that the feature contributes positively to the prediction, while negative values indicate a negative contribution. The SHAP values provide a quantitative measure of the contribution of each feature to the model's output, enabling a more interpretable understanding of how individual features influence the algorithm's decision-making process. This transparency is crucial for building trust in AI systems and facilitating their use in various real-world applications where interpretability is essential (Janzing et al., 2020). This approach contributes to the trustworthiness and applicability of our findings, enhancing the overall validity of the study's outcomes in the context of Mozzarella di Bufala PDO from Salerno and Caserta.

## 4 Results

This study aims to investigate the potential use of explainable artificial intelligence for identifying the food origin. The case of study of Mozzarella di Bufala Campana PDO has been considered by examining the composition of the microbiota in 65 samples.

This study involved evaluating the effectiveness of three supervised machine learning algorithms, namely XGBoost, Random Forest, and a complex Multi-Layer Perceptron network. The analysis revealed that the Random Forest classifier outperformed the others, demonstrating the highest Area Under the Curve (AUC) value of 0.93 ± 0.10 and the top accuracy score of 0.87 ± 0.11. Table 2 provides a comprehensive comparison of the three models based on their AUC and accuracy scores.

**Table 2:** Comparison between evaluation metrics of XGBoost (XGB), Random Forest (RF), and Multi-Layer Perceptron (MLP) classifiers.

**Classifier**	**Accuracy**	**AUC**
XGB	0.82 ± 0.12	0.87 ± 0.11
RF	0.87 ± 0.11	0.93 ± 0.10
MLP	0.68 ± 0.13	0.78 ± 0.11

### 4.1 Machine learning analysis

**Figure 2:**
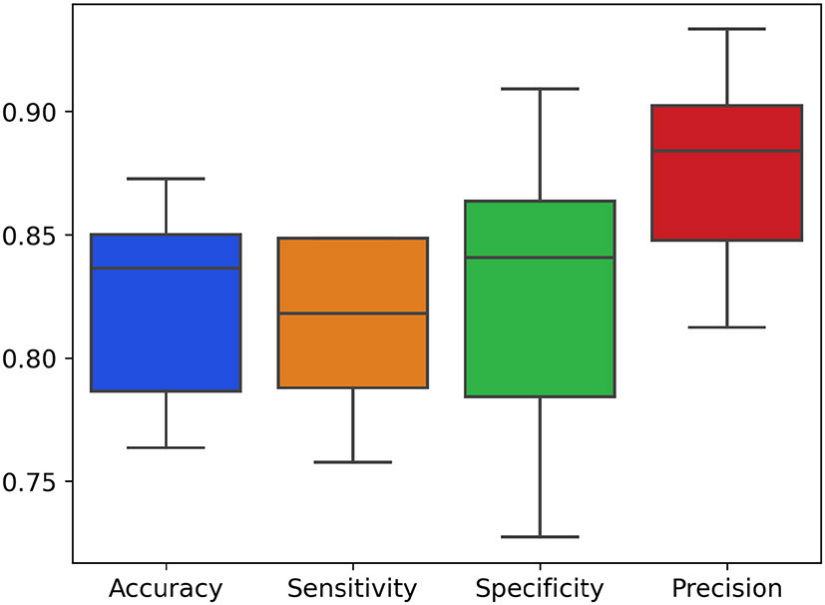
Boxplot of the distributions of evaluation metrics (accuracy, specificity, sensitivity and precision) following five-fold cross-validation repeated 20 times.

The results are illustrated in the confusion matrix in Table 3, obtained following a five-fold repeated 20 times cross-validation procedure on the validation set. This methodology allows us to assess the effectiveness of our algorithm in a robust and reliable manner. In Figure 2 it is possible to observe the boxplot displaying the trend evaluation metrics, including accuracy (Equation 1), specificity (Equation 3), sensitivity (Equation 2) and precision (Equation 4), obtained through a five-fold repeated cross-validation scheme.

**Table 3:** Confusion matrix depicts predicted values against actual values.

**Actual class**		**Predicted class**	
		**Caserta**	**Salerno**
	Caserta	29	4
	Salerno	3	19

In this instance, 29 samples from Caserta and 19 from Salerno are correctly classified, while four samples from Caserta and three from Salerno are misclassified.

The confusion matrix highlights the algorithm's ability to correctly classify observations based on the geographical origin of the samples, divided between the Salerno and Caserta areas. We observe that the algorithm achieved an accuracy of 87.87% in correctly identifying samples from the Salerno area and 86.36% for those from the Caserta area. These results indicate a good capability of our machine learning model in distinguishing the geographical origin of Mozzarella di Bufala Campana PDO based on the microbiota structure. The accuracy in both cases is quite high, suggesting that the model generalizes well to new data and could be used as a supportive tool in determining the geographical origin of unknown samples.

**Figure 3:**
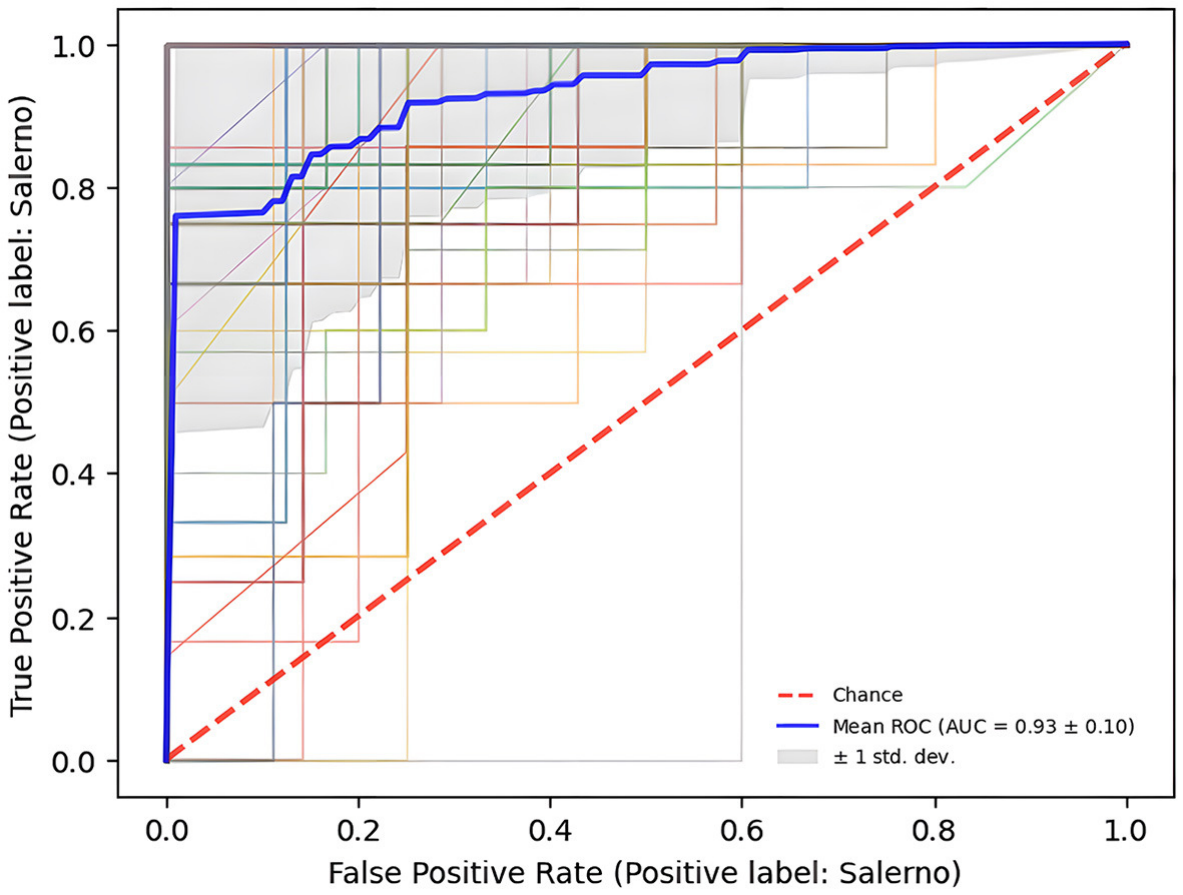
ROC curve depicts the classification model's ability to vary the trade-off between sensitivity (True Positive Rate) and specificity (1 – False Positive Rate).

The Receiver Operating Characteristic curve in the Figure 3 defines AUC score, measuring the area under this curve, is 0.93 ± 0.10 and it suggests a high accuracy in classifying samples based on their geographical origin, affirming the robustness of the model's performance.

**Figure 4:**
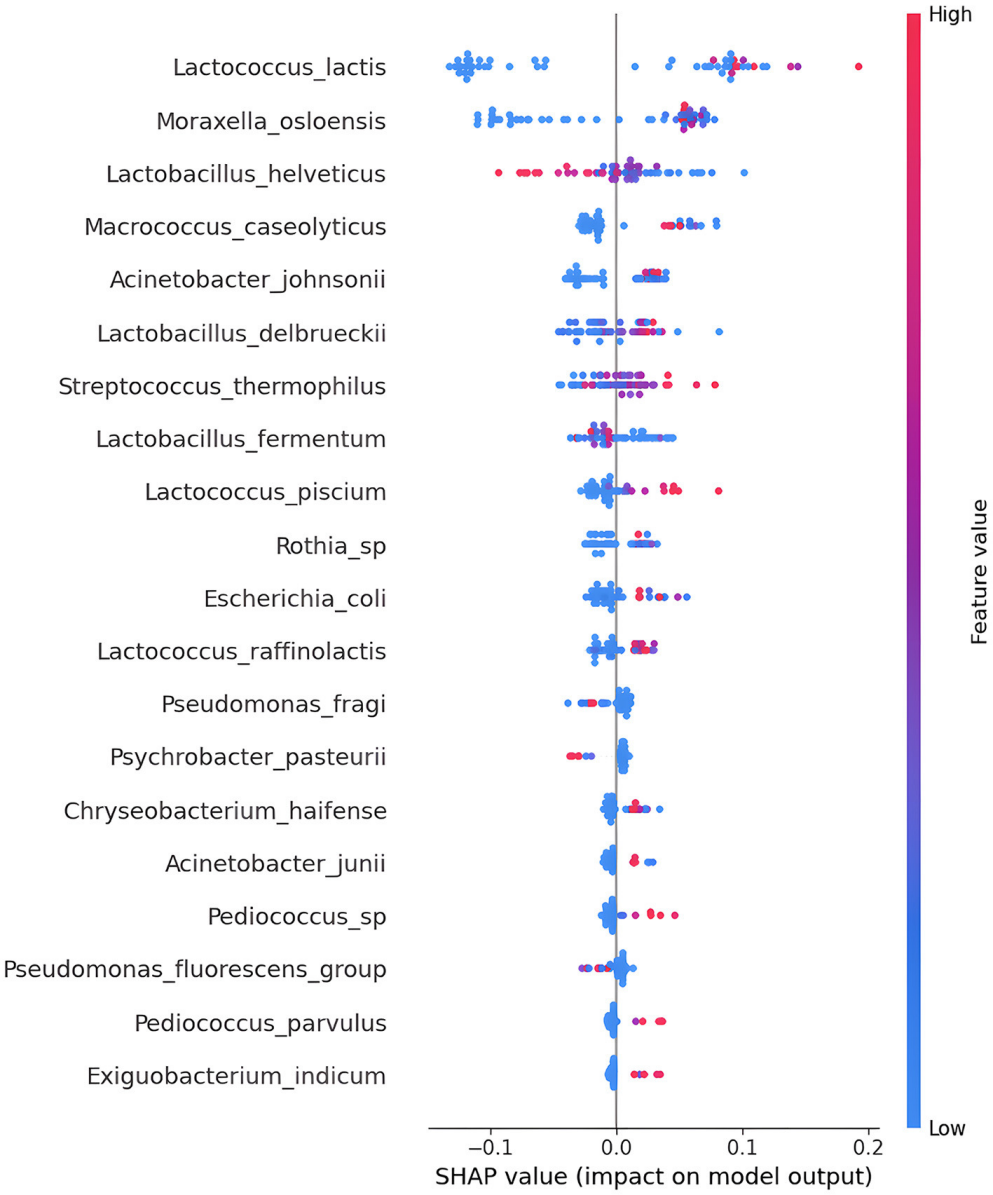
The SHapley Additive exPlanations (SHAP) summary plot provides an overview of the importance of features in contributing to model predictions. In this type of plot, each point represents a data instance, and the horizontal position of the point indicates how much the effect of a specific feature contributes to the change in prediction compared to the model's average prediction. The color of the point represents the value of the feature, with darker colors indicating higher values.

After conducting cross-validation, the outcomes were then utilized to compute feature importance employing SHapley Additive exPlanations (SHAP), as expressed in Equation (5). The SHAP ranking plot is a graph that displays the importance of features in machine learning models using SHAP and features are arranged along the y-axis based on their importance, with the most important features at the top and the least important ones at the bottom. Each colored point represents a single data instance, and the horizontal position of the point indicates the value of the shap for that specific instance. The color of the point indicates the value of the feature: higher values are represented in warm colors (red), while lower values are represented in cool colors (blue). Through a SHAP analysis, the 20 most important feature were identified, deriving from the analysis of the microbiota 65 samples. In the SHAP plot in Figure 4 it is evident how certain features, such as *Lactococcus lactis* and *Moraxella osloensis*, contribute significantly to the model's prediction. The feature *Lactobacillus helveticus* is important for the model's interpretability, as the colored points are well distinguished, and red points indicate that high values of that bacterium have influenced Salerno class, and vice versa. This suggests that these elements play a crucial role in the geographical discrimination of the samples.

The results of the Shap analysis highlight the fact that two Phyla are most represented (Firmicutes and Proteobacteria). The taxonomy of each sample of SHAP analysis is descripted in Table 4. Lactobacillaceae is represented by five bacteria, Moraxella family is represented by four bacteria, while Lactococcaceae family is represented by three bacteria. Starting from the taxonomic group of the genus, it can be seen that there is a significant diversity of microbes, even if the *Lactococcus* genus and *Lacotbacillus* genus is represented three times each other.

**Table 4:** Classification of the first 20 bacteria deriving from the Shap analysis.

**Phylum**	**Class**	**Order**	**Family**	**Genus**	**Species**
Firmicutes	*Bacilli*	*Lactobacillales*	*Lactococcaceae*	*Lactococcus*	*Lactococcus lactis*
Proteobacteria	*Gammaproteobacteria*	*Pseudomonadales*	*Moraxellaceae*	*Moraxella*	*Moraxella osloensis*
Firmicutes	*Bacilli*	*Lactobacillales*	*Lactobacillaceae*	*Lactobacillus*	*Lactobacillus helveticus*
Firmicutes	*Bacilli*	*Bacillales*	*Staphylococcaceae*	*Macrococcus*	*Macrococcus caseolyticus*
Proteobacteria	*Gammaproteobacteria*	*Pseudomonadales*	*Moraxellaceae*	*Acinetobacter*	*Acinetobacter johnsonii*
Firmicutes	*Bacilli*	*Lactobacillales*	*Lactobacillaceae*	*Lactobacillus*	*Lactobacillus delbrueckii*
Firmicutes	*Bacilli*	*Lactobacillales*	*Streptococcaceae*	*Streptococcus*	*Streptococcus thermophilus*
Firmicutes	*Bacilli*	*Lactobacillales*	*Lactobacillaceae*	*Lactobacillus*	*Lactobacillus fermentum*
Firmicutes	*Bacilli*	*Lactobacillales*	*Lactococcaceae*	*Lactococcus*	*Lactococcus piscium*
Actinobacteria	*Actinobacteria*	*Micrococcales*	*Micrococcaceae*	*Rothia*	*Unclassified bacterium*
Proteobacteria	*Gammaproteobacteria*	*Enterobacterales*	*Enterobacteriaceae*	*Escherichia*	*Escherichia coli*
Firmicutes	*Bacilli*	*Lactobacillales*	*Lactococcaceae*	*Lactococcus*	*Lactococcus raffinolactis*
Proteobacteria	*Gammaproteobacteria*	*Pseudomonadales*	*Pseudomonadaceae*	*Pseudomonadaceae*	*Pseudomonadaceae fragi*
Proteobacteria	*Gammaproteobacteria*	*Pseudomonadales*	*Moraxellaceae*	*Psychrobacter*	*Psychrobacter pasteurii*
Bacteroidetes	*Flavobacteriia*	*Flavobacteriales*	*Weeksellaceae*	*Chryseobacterium*	*Chryseobacterium haifense*
Proteobacteria	*Gammaproteobacteria*	*Pseudomonadales*	*Moraxellaceae*	*Acinetobacter*	*Acinetobacter junii*
Firmicutes	*Bacilli*	*Lactobacillales*	*Lactobacillaceae*	*Pediococcus*	*Unclassified bacterium*
Proteobacteria	*Gammaproteobacteria*	*Pseudomonadales*	*Pseudomonadaceae*	*Pseudomonadaceae*	*Pseudomonadaceae fluorescens*
Firmicutes	*Bacilli*	*Lactobacillales*	*Lactobacillaceae*	*Pediococcus*	*Pediococcus parvulus*
Firmicutes	*Bacilli*	*Bacillales*	*Bacillaceae*	*Exiguobacterium*	*Exiguobacterium indicum*

The Phylum, Class, Order, Family, Genus and Species columns indicate the classification of each bacteria.

A possible application of the classification model is to execute it on the previously selected test dataset. In testing the model, a dataset consisting of 10 samples from the same study was utilized, including two from Caserta and eight from Salerno. These samples were previously excluded during the model training phase. The confusion matrix of the test, depicted in the figure, provides a detailed overview of the model's performance on this specific test dataset. It is particularly noteworthy that all samples from Caserta were correctly classified by the model. On the other hand, only one sample from Salerno was misclassified. This result suggests a significant accuracy in the model's ability to discriminate between the two production locations, with a particularly high success rate for samples from Caserta. The confusion matrix in Table 5 offers a detailed assessment of the model's performance on the specific test dataset.

**Table 5:** Confusion matrix depicts predicted values against actual values.

**Actual class**		**Predicted class**	
		**Caserta**	**Salerno**
	Caserta	2	0
	Salerno	1	7

In this instance, seven samples from Salerno and two from Caserta are correctly classified, while only one sample from Salerno is misclassified.

## 5 Discussion

Mozzarella di Bufala Campana PDO is a designation that certifies the mozzarella is produced in the Campania region, Italy, and follows traditional production methods and established quality standards to preserve its authenticity and excellence. The PDO protects the product name from imitations and assures buyers that they are purchasing a genuine product produced according to the traditional specifications of the designated area. Recognizing the correct origin is crucial to preserving the diversity and excellence of local productions. Protection against imitations and counterfeits, guaranteed by the PDO, helps maintain the product's reputation and preserves its cultural history. Ultimately, correctly identifying the origin of PDO mozzarella not only ensures product quality but also contributes to preserving the cultural and gastronomic heritage associated with this unique Italian specialty.

Indeed, the integration of machine learning (ML) and explainable artificial intelligence (XAI) techniques holds significant value in various contexts, including the analysis of biological data such as microbiota and metabolomics. Machine learning facilitates the creation of accurate predictive models based on microbiological data, aiding in the authentication and protection of PDO products like Mozzarella di Bufala Campana. XAI techniques ensure transparency and interpretability, reinforcing trust among consumers, regulators, and industry stakeholders. This combination not only enhances the certification of food origin but also strengthens the preservation of cultural and gastronomic heritage associated with traditional foods. Overall, microbiota analysis plays a vital role in ensuring the authenticity, quality, and safety of food products like Mozzarella di Bufala Campana PDO. In this study, each sample exhibits a relative abundance of various microbial species, which are not present in all samples. The most prevalent genera are *Pseudomonas, Lactobacillus, Streptococcus*, and *Acinetobacter*. The cheese-making process of Mozzarella di Bufala Campana is a combination of high-quality ingredients and specific procedures, with particular attention to the crucial role played by natural whey containing thermophilic lactic bacteria. The presence of thermophilic lactic bacteria is interesting because they survive at high temperatures during the processing, thus contributing to the uniqueness of Mozzarella di Bufala Campana (Levante et al., 2023). The ecological complexity of these thermophilic lactic bacteria is an aspect that can be studied in detail to better understand the fermentation process and the production of this traditional cheese. Research conducted has shown that, despite ecological complexity, only certain thermophilic lactic acid bacteria (LAB), namely *Streptococcus thermophilus, Lactobacillus delbrueckii*, and *Lactobacillus helveticus*, are the main players in the curd fermentation. This is one of the peculiarities that helps preserve the unique characteristics of the cheese and protects local producers from imitations and counterfeits. It also assures buyers that they are purchasing an authentic and high-quality product, respecting the long history and reputation of Mozzarella di Bufala Campana as a traditional and artisanal product (Pisano et al., 2016).

## 6 Conclusion

This paper is an example of how an XAI analysis can be applied with trustworthiness in the context of discriminating the geographical origin of PDO Mozzarella di Bufala Campana based on microbiota bacterial abundance. This validates the approach employed in our study and confirms that certain bacteria can be considered reliable indicators of geographical origin. The predictive models developed using machine learning techniques have proven to be effective in classifying the geographical origin of mozzarella samples. These results provides strong support for food traceability, enabling consumers to make informed choices and ensuring that products are authentic and safe. The results obtained have significant implications for the food industry as they offer an innovative and reliable method to authenticate and protect high-quality regional products. This can contribute to strengthening consumer confidence in food products and supporting local economies through the promotion of sustainable agricultural practices. Further research could delve deeper into microbiota analysis and assess the effectiveness of other analytical techniques in improving the accuracy of predictions regarding the geographical origin of food products. Machine learning facilitates the creation of robust predictive models capable of accurately identifying the origin of food products based on microbiological data. Furthermore, XAI techniques provide transparency and interpretability, enabling stakeholders to understand how these models arrive at their conclusions. This combination not only ensures the trustworthiness of predictions but also fosters trust among consumers, regulators, and industry professionals. Moving forward, further research could delve deeper into microbiota analysis and explore the effectiveness of additional analytical techniques in enhancing the accuracy of predictions regarding the geographical origin of food products. Additionally, investigating the application of these approaches in diverse contexts and food products would expand the scope and applicability of our findings, driving continual advancements in food traceability and quality assurance practices.

## Data availability statement

The data presented in the study are deposited in the Sequence Read Archive (SRA) database of the NCBI, accession numbers PRJNA1084214 and PRJNA997821.

## Author contributions

MM: Writing – review & editing, Writing – original draft, Software, Methodology, Investigation, Formal analysis. PN: Writing – review & editing, Writing – original draft, Visualization, Validation, Methodology, Investigation, Conceptualization. FD: Writing – review & editing, Validation, Investigation, Data curation. RM: Writing – review & editing, Data curation. PD: Writing – review & editing, Validation. DD: Writing – review & editing, Validation. RB: Writing – review & editing, Validation. ST: Writing – review & editing, Writing – original draft, Validation, Supervision, Project administration, Methodology, Investigation, Funding acquisition, Conceptualization.
